# Research on Economic Optimization of Microgrid Cluster Based on Chaos Sparrow Search Algorithm

**DOI:** 10.1155/2021/5556780

**Published:** 2021-03-10

**Authors:** Peng Wang, Yu Zhang, Hongwan Yang

**Affiliations:** College of Mechanical and Control Engineering, Guilin University of Technology, Guilin 541000, China

## Abstract

With the deepening of the power market reform on the retail side, it is of great significance to study the economic optimization of the microgrid cluster system. Aiming at the economics of the microgrid cluster, comprehensively considering the degradation cost of energy storage battery, the compensation cost of demand-side controllable loads dispatch, the electricity transaction cost between the microgrids, and the electricity transaction cost between the microgrid and the power distribution network of the microgrid cluster, we establish an optimal dispatch model for the microgrid cluster including wind turbines, photovoltaics, and energy storage batteries. At the same time, in view of the problem that the population diversity of the basic sparrow search algorithm decreases and it is easy to fall into local extremes in the later iterations of the basic sparrow search algorithm, a chaos sparrow search algorithm based on Bernoulli chaotic mapping, dynamic adaptive weighting, Cauchy mutation, and reverse learning is proposed, and different types of test functions are used to analyze the convergence effect of the algorithm, and the calculation effects of the sparrow algorithm, the particle swarm algorithm, the chaotic particle swarm, and the genetic algorithm are compared. The algorithm has higher convergence speed, higher accuracy, and better global optimization ability. Finally, through the calculation example, it is concluded that the benefit of the microgrid cluster is increased by nearly 20%, which verifies the effectiveness of the improvement.

## 1. Introduction

With the current energy shortage and environmental problems in power supply becoming more and more serious, the microgrid composed of renewable energy sources has been widely used [1–3], which can not only improve the energy efficiency but also protect the environment, reduce costs, and meet the requirements of economy, environmental protection, and stability [4–10]. However, due to the poor antidisturbance capacity of a single microgrid, multiple microgrids in a local area are interconnected to form a microgrid cluster system. By coordinating the energy output of each microgrid in the cluster, the dynamic balance of supply and demand within the cluster is achieved, and the reliability and economy of the system is effectively improved. However, due to differences in the capacity configuration and load characteristics of each microgrid, when electric energy is exchanged between microgrids, it will affect the operation of microgrids [11]. Therefore, considering the exchange of electricity between microgrids, how to coordinate and optimize the microgrid cluster according to the supply and demand characteristics of each microgrid is very important.

At present, research on coordination and optimization of a microgrid cluster has achieved certain results. Literature [12] proposed a multi-microgrid coordinated and optimized dispatching model based on double auction to optimize the sum of interactive power between each microgrid and other microgrids and power distribution network. Although this model optimized the electricity transaction volume between microgrids, it did not take into account the influence of demand-side controllable loads on the electrical energy interaction between microgrids. Literature [13] established a market transaction model based on cooperative game theory and carried out research on the benefit distribution and settlement rules of the microgrid cluster cooperative alliance. Although the electricity transaction price between microgrids was optimized, the influence of the demand-side controllable loads on the microgrids was not considered. Literature [14] studied the optimal dispatching problem of photovoltaic microgrid based on the consideration of time-of-use electricity price, with the goal of minimizing the exchange volume of the grid. Although the time-of-use electricity prices and optimized transaction volumes were considered, only the optimal dispatch of a single microgrid was considered, and the optimization of the microgrid cluster system was not considered. Literature [15] considered the installation cost, operating cost, and environmental benefits of the multi-microgrid system to establish an economic dispatch model and used a particle swarm optimization algorithm combined with Monte Carlo simulation to solve it. Although a centralized optimization algorithm was adopted, each microgrid needed to fully share internal privacy information, which requires higher communication and makes it difficult to optimize dispatch. Literature [16] achieved minimum operation cost through a few iterations by combining the Newton-type second-order algorithm and a consensus-based information exchange. Literature [17] took the adjustment cost of each microgrid as the consensus variables and used the consensus algorithm to solve the power distribution problem of island microgrid clusters. Although literature [16] and [17] had realized the economic operation of microgrid clusters, they only considered peak shaving and valley filling from the power generation side, without considering the response effect of electricity load. Literature [18] was based on the particle swarm algorithm to study the economic optimization dispatch of the microgrid. Although the optimal solution of the microgrid operating cost was obtained, the particle swarm algorithm had limited global optimization capability and is easy to fall into the local optimal solution. Literature [19] proposed a chaotic starling particle swarm optimization algorithm. The additions of the inertial weights and the chaotic logistic mapping strategy improved the algorithm to have better convergence and stronger global search capabilities. However, when applied to multiple local optima, it may fall into local optima and cause stagnation. Literature [20] proposed a new type of swarm intelligence optimization algorithm, the sparrow search algorithm. Compared with other algorithms, its solution efficiency was better; however, it was also easy to fall into the problem of local extremum in the later iteration.

Based on the abovementioned problems, this paper improves the optimization model of the microgrid cluster and comprehensively considers the degradation cost of energy storage battery, the compensation cost of demand-side controllable loads dispatch, the electricity transaction cost between the microgrids, and the electricity transaction between the microgrid and the power distribution network of the microgrid cluster, establishes a dynamic energy trading model for the microgrid cluster, promotes the balance of supply and demand within the cluster by continuously coordinating the electricity transaction volume between microgrids, and reduces system operating costs. At the same time, taking into account the Bernoulli chaotic map, dynamic adaptive weights can effectively avoid the algorithm from falling into the local optimum and improve the algorithm's global optimization ability, and the Cauchy mutation and reverse learning can effectively jump out of the local optimum. This paper proposes a chaotic sparrow search algorithm (ISSA). We use different types of test functions to analyze the convergence effect of the algorithm and compare them with sparrow algorithm (SSA), particle swarm algorithm (PSO), chaotic particle swarm algorithm (CPSO), and genetic algorithm (GA). The effectiveness of the improvement of the proposed ISSA algorithm is verified, and the algorithm is applied to solve the operating cost of the microgrid cluster system. Finally, a calculation example is used to verify the operating economy of the microgrid cluster with demand-side controllable loads participating in the dispatch.

The organization structure of this paper is as follows.  Section 2 introduces the basic structure of the microgrid cluster and demand-side response model.  In Section 3, the optimal dispatch model of the microgrid cluster is introduced.  In Section 4, the improved sparrow algorithm is introduced.  Section 5 uses the test function analysis to verify the effectiveness of the algorithm improvements.  In Section 6, the microgrid cluster composed of three microgrids is taken as an example, and the ISSA algorithm is used to conduct simulation experiments.  Section 7 gives the conclusion and describes the future of the proposed algorithm.

## 2. Microgrid Cluster Structure and Demand-Side Response Model

### 2.1. Electric Energy Trading Structure of the Microgrid Cluster

**Figure 1:**
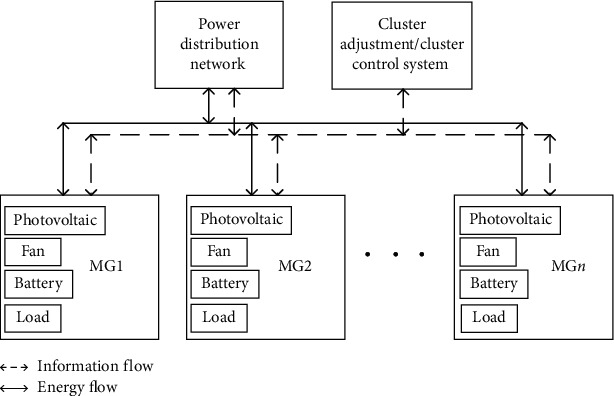
Electricity transaction of the microgrid cluster.

The microgrid cluster electric energy transaction structure is shown in Figure 1; *n* microgrids are interconnected to form a microgrid cluster system, and each microgrid in the community contains different types of distributed power generation units and loads. The energy flow in Figure 1 represents the power interaction between the microgrid and the power distribution network, and the information flow represents the two-way interaction of information between the microgrid and the cluster adjustment/cluster control system. It can be seen from Figure 1 that, in the process of optimal dispatch of the microgrid cluster, the cluster adjustment/cluster control system conducts two-way information interaction with the microgrid and continuously optimizes the electric energy transaction volume by adding controllable loads to realize the optimal dispatch of the microgrid cluster.

### 2.2. Demand-Side Response Model

There are many different forms of loads in the microgrid. The classification of loads is helpful for energy management and dispatch. The demand-side management model of this paper mainly takes controllable loads as the research object. Based on the reliability of power supply, it is divided into three types: shiftable load, transferable load, and interruptible load.

#### 2.2.1. Shiftable Load

The microgrid can flexibly change the operating period of the shiftable load, so that it can choose to use electricity in the appropriate electricity price time period. The microgrid can control the shiftable load from the peak period of power consumption to the general period, thereby reducing the pressure of dispatching and reducing the operating cost. The mathematical model is as follows [21]:

(1) $$\begin{array}{c} C_{i,k,t}^{\text{shift}}=C_{i}^{P_{\text{shift}}}P_{i,k}^{\text{shift}}d_{i,k,t}, \end{array}$$

where *C*_*i*_^*P*_shift_^ represents the dispatch compensation cost of unit power of the shiftable load in MGi, *P*_*i*,*k*_^shift^ represents the power of the *k*-th translatable load in MGi, *d*_*i*,*k*,*t*_^shift^ represents the state of the *k*-th translatable load of MGi in period *t*, if *t* ∈ [*t*_*i*,*k*_^shift^, *t*_*i*,*k*_^shift^+*D*_*i*,*k*_^shift^], then when the value is 1, it means it is in the running state; when the value is 0, it means it is in the interrupt state, *t*_*i*,*k*_^shift^ represents the original power consumption period of the *k*-th shiftable load in MGi, and *D*_*i*,*k*_^shift^ represents the continuous power consumption period of the *k*-th shiftable load in MGi.

#### 2.2.2. Transferable Load

The transferable load has a certain degree of flexibility in the way of power supplies, its controllability is very strong, and the power supply time can be changed as planned. The power sector estimates the transferable load capacity and signs contracts with users to increase the economy of the microgrid. The mathematical model is as follows:

(2) $$\begin{array}{c} C_{i,k,t}^{\text{trans}}=C_{i}^{P_{\text{trans}}}P_{i,k}^{\text{trans}}u_{i,k,t}, \end{array}$$

where *C*_*i*_^*P*_trans_^ represents the dispatch compensation cost of unit power of the shiftable load in MGi, *P*_*i*,*k*_^trans^ represents the power of the *k*-th transferable load in MG I, *u*_*i*,*k*,*t*_ represents the state of the *k*-th transferable load of the MGi in the period *t*, if *t* ∈ [*t*_*i*,*k*_^trans^, *t*_*i*,*k*_^trans^+*D*_*i*,*k*_^trans^], then when the value is 1, it means it is in the running state; when the value is 0, it means it is in the interrupt state, *t*_*i*,*k*_^*P*_trans_^ represents the original power consumption period of the *k*-th shiftable load in MGi, and *D*_*i*,*k*_^trans^ represents the total power consumption period of the *k*-th shiftable load in MGi.

#### 2.2.3. Interruptible Load

The microgrid can cut off the interruptible load at any time without any negative impact on its operation, thus making the microgrid's energy management system more flexible. Cutting off the interruptible load requires consideration of the user's wishes and carries on certain compensation to the user. When the compensation cost for removing the interruptible load is lower than the cost of dispatching other units, it can choose to remove the interruptible load. The mathematical model is as follows:

(3) $$\begin{array}{c} C_{i,k,t}^{\text{inter}}=\left(1-f_{i,k,t}\right)C_{i}^{P_{\text{inter}}}P_{i,k}^{\text{inter}}, \end{array}$$

where *f*_*i*,*k*,*t*_ represents the state of the *k*-th interruptible load of the MGi in the *t* period, when the value is 1, it means it is in the running state, when the value is 0, it means it is in the interrupt state, and *C*_*i*_^*P*_inter_^ and *P*_*i*,*k*_^inter^, respectively, represent the dispatch compensation cost of unit power of the interrupted load in MGi and the power of the *k*-th interruptible load.

When the user side participates in demand management, the power department should provide economic compensation to the user. This article comprehensively considers the dispatch compensation cost of the three controllable loads: shiftable load, transferable load, and interruptible load. The specific mathematical model is as follows:

(4) $$\begin{array}{c} C_{i,t}^{\text{CL}}=\sum_{i\in N_{i}^{\text{shift}}}C_{i,k,t}^{\text{shift}}+\sum_{i\in N_{i}^{\text{trans}}}C_{i,k,t}^{\text{trans}}+\sum_{i\in N_{i}^{\text{inter}}}C_{i,k,t}^{\text{inter}}, \end{array}$$

where *C*_*i*,*k*,*t*_^shift^ represents the dispatch compensation cost of the *k*-th shiftable load of MGi in period *t*, *C*_*i*,*k*,*t*_^trans^ represents the dispatch compensation cost of the *k*-th shiftable load of MGi in period *t*, *C*_*i*,*k*,*t*_^inter^ represents the dispatch compensation cost of the *k*-th interruptible load of MGi in period *t*, *N*_*i*_^shift^ represents the set of translatable load of MGi, *N*_*i*_^trans^ represents the set of transferable load of MGi, and *N*_*i*_^inter^ represents the set of interruptible load of MGi.

## 3. Optimal Dispatch Model of the Microgrid Cluster

### 3.1. Operating Cost Model

#### 3.1.1. Degraduation Cost of the Energy Storage Battery

The degradation cost of the energy storage battery is the cost of life reduction caused by the battery being recycled due to discharge, which is determined by the depth of discharge and the price of the battery. When the energy storage battery is deeply discharged, its recycling will lead to a reduction in life and an increase in degradation costs. In this paper, the battery discharge depth function [22] is used to express the degradation cost of the energy storage battery. The specific function can be expressed as

(5) $$\begin{array}{c} \left\{\begin{array}{c} C_{i,t}^{\text{BD}}=\left(10^{-\left(S_{i}^{\max}-S_{i,t}\right)}-10^{-\left(S_{i}^{\max}-S_{i,t-1}\right)}\right)C_{i,\text{bat}},\\ S_{i,t}=S_{i,t-1}+\frac{P_{i,t}^{\text{ch}}\Delta t\eta_{i,t}^{\text{ch}}}{N_{i}^{\text{ESS}}}-\frac{P_{i,t}^{\text{dis}}\Delta t}{N_{i}^{\text{ESS}}\eta_{i,t}^{\text{dis}}}, \end{array}\right) \end{array}$$

where *S*_*i*_^max^ represents the maximum state of charge of the energy storage battery of MGi, *S*_*i*,*t*_ represents the state of charge of the energy storage battery of MGi in the period *t*, *C*_*i*,bat_ represents the initial investment cost of the energy storage battery of MGi, *P*_*i*,*t*_^ch^ represents the charging power of the energy storage battery of the MGi in the period *t*, *P*_*i*,*t*_^dis^ represents the discharge power of the energy storage battery of the MGi in the period *t*, *N*_*i*_^ESS^ represents the rated capacity of the energy storage battery of the MGi, *η*_*i*,*t*_^ch^ represents the charging efficiency of the energy storage battery of the MGi in the period *t*, *η*_*i*,*t*_^dis^ represents the discharge efficiency of the energy storage battery of the MGi in the period *t*, and Δ*t* is the time interval.

From equation (5), it can be seen that the initial investment cost of the energy storage battery is constant. Therefore, as long as the charge and discharge power of the energy storage battery are optimized to obtain the battery state of charge, the degradation cost of the energy storage battery can be obtained.

#### 3.1.2. Electricity Transaction Cost between Microgrids

The electricity transaction cost between microgrids refers to the cost incurred when multiple microgrids exchange electricity, which is generally expressed as

(6) $$\begin{array}{c} C_{t,k}^{\text{MN}}=P_{i,t}^{\text{MN}}\lambda_{i,t}^{\text{MN}}\Delta t, \end{array}$$

where *P*_*i*,*t*_^MN^ represents the power of MGi interacting with other microgrids in period *t*, the purchase of electricity is a positive value, the sale of electricity is a negative value, and *λ*_*i*,*t*_^MN^ represents the electricity price of MGi in period *t*.

#### 3.1.3. Electricity Transaction Cost between the Microgrid and Power Distribution Network

The electricity transaction cost between the microgrid and the distribution network refers to the cost generated when the microgrid and the power distribution network interact with electricity, which is generally expressed as

(7) $$\begin{array}{c} C_{i,t}^{\text{DN}}=-P_{i,t}^{\text{DN}}\lambda_{t}^{\text{DN}}\Delta t, \end{array}$$

where *P*_*i*,*t*_^DN^ is the interactive power between the MGi and the power distribution network in *t* period, the purchase of electricity is a negative value, the sale of electricity is a positive value, and *λ*_*i*,*t*_^DN^ is the electricity price of the power distribution network in *t* period.

### 3.2. Optimal Dispatch Objective Function of the Microgrid Cluster

This paper takes the lowest operating cost of the microgrid cluster as the optimization goal and establishes the optimal dispatch model of the microgrid cluster. During the operation of the microgrid cluster, the costs are mainly composed of the compensation cost of the demand-side controllable load dispatch, the degradation cost of the energy storage battery, the electricity transaction cost between the microgrids, and the electricity transaction cost between the microgrid and power distribution network. The optimization objective function can be expressed as

(8) $$\begin{array}{c} \min f\left(x\right)=\sum_{t\in T}C_{i,t}^{\text{CL}}+\sum_{t\in T}C_{i,t}^{\text{BD}}+\sum_{t\in T}C_{i,t}^{\text{MN}}+\sum_{t\in T}C_{i,t}^{\text{DN}}, \end{array}$$

where *C*_*i*,*t*_^CL^ represents the dispatch compensation cost of the controllable loads of the MGi in period *t*, *C*_*i*,*t*_^BD^ represents the degradation cost of the energy storage battery of the MGi in period *t*, *C*_*i*,*t*_^MN^ represents the electricity transaction cost between the microgrids of the MGi in period *t*, *C*_*i*,*t*_^DN^ represents the electricity transaction cost between the microgrid and the power distribution network of MGi in period *t*, and *T* is the dispatch period.

### 3.3. Constraints

#### 3.3.1. Constraints of Power Balance

The power balance constraint of the microgrid clusters system is generally expressed as

(9) $$\begin{array}{c} P_{i,t}^{\text{DN}}\;+P_{i,t}^{\text{IL}}+P_{i,t}^{\text{ch}}+\sum_{k\in N_{i}^{\text{shift}}}P_{i,k}^{\text{shift}}d_{i,k,t}+\sum_{k\in N_{i}^{\text{rans}}}P_{i,k}^{\text{trans}}u_{i,k,t}+\sum_{k\in N_{i}^{\text{inter}}}P_{i,k}^{\text{inter}}f_{i,k,t}=P_{i,t}^{\text{RE}}+P_{i,t}^{\text{dis}}+P_{i,t}^{\text{MN}}, \end{array}$$

where *P*_*i*,*t*_^IL^ is the electric power of the key load of MGi in *t* period and *P*_*i*,*t*_^RE^ is the renewable energy output of MGi in *t* period.

The interactive power balance constraint between microgrids is expressed as

(10) $$\begin{array}{c} \sum_{i\in I}P_{i,t}^{\text{MN}}=0. \end{array}$$

#### 3.3.2. Constraints of Energy Storage Battery Operation

Since the energy storage battery cannot be charged and discharged at the same time, the energy storage battery charge and discharge power constraint is expressed as

(11) $$\begin{array}{c} \left\{\begin{array}{c} 0\leq P_{i,t}^{\text{ch}}\leq P_{i}^{\text{max}},\\ 0\leq P_{i,t}^{\text{dis}}\leq P_{i}^{\text{max}},\\ P_{i,t}^{\text{ch}}\cdot P_{i,t}^{\text{dis}}=0, \end{array}\right) \end{array}$$

where *P*_*i*_^max^ represents the maximum charge and discharge power of the energy storage battery of the MGi.

For energy storage battery, their initial and final capacities should be consistent (excluding charge and discharge power constraints) and the energy storage capacity of each period needs to be controlled within a reasonable range. The following is the specific expression:

(12) $$\begin{array}{c} \left\{\begin{array}{c} S_{i}^{\min}\leq S_{i,t}\leq S_{i}^{\max},\\ S_{i,0}=S_{i,24}, \end{array}\right) \end{array}$$

where *S*_*i*_^min^ is the minimum state of charge of the energy storage battery of MGi.

#### 3.3.3. Transaction Constraints of the Microgrid Cluster

(1) Trading electricity price constraints between microgrids: when directly trading electrical energy between microgrids, the price should be above the purchase price of the power distribution network and remain below the sale price of the power distribution network, so it is expressed as

(13) $$\begin{array}{c} \lambda_{t}^{\text{EB}}\leq \lambda_{i,t}^{\text{MN}}\leq \lambda_{t}^{\text{ES}}, \end{array}$$

where *λ*_*t*_^EB^ and *λ*_*t*_^ES^ are the electricity purchase and sale prices of the power distribution network.

(2) Constraints on the balance of electricity transaction costs between microgrids: the balance constraint of electricity transaction cost between microgrids is expressed as

(14) $$\begin{array}{c} \sum_{i\in I}P_{i,t}^{\text{MN}}\lambda_{i,t}^{\text{MN}}\Delta t=0. \end{array}$$

(3) Interactive power constraints between microgrids: the interactive power constraint between microgrids is expressed as

(15) $$\begin{array}{c} -P_{\max}^{\text{MN}}\leq P_{i,t}^{\text{MN}}\leq P_{\max}^{\text{MN}}, \end{array}$$

where *P*_max_^MN^ is the maximum value of interactive power between microgrids.

(4) Interactive power constraints between the microgrid and power distribution network: the interactive power constraint between the microgrid and the power distribution network is expressed as

(16) $$\begin{array}{c} -P_{\max}^{\text{DN}}\leq P_{i,t}^{\text{DN}}\leq P_{\max}^{\text{DN}}, \end{array}$$

where *P*_max_^DN^ is the maximum value of the interactive power between the MGi and the power distribution network.

#### 3.3.4. Constraints of Demand-Side Controllable Loads

The shiftable load needs to be translated as a whole to maintain the continuity of its electricity consumption, so the shiftable load constraint is expressed as

(17) $$\begin{array}{c} \left\{\begin{array}{c} \sum_{t=t_{i,k}^{S_{\text{shift}}}}^{t_{i,k}^{E_{\text{shift}}}}d_{i,k,t}=D_{i,k}^{\text{shift}},\\ d_{i,k,t}+\sum_{p=1}^{D_{i,k}^{\text{shift}}-1}d_{i,k,t+p}=D_{i,k}^{\text{shift}}, \end{array}\right) \end{array}$$

where *t*_*i*,*k*_^*S*_shift_^ represents the initial dispatch period of the *k*-th shiftable load in MGi and *t*_*i*,*k*_^*E*_shift_^ represents the end dispatch period of the *k*-th shiftable load in MGi.

The dispatch period of the transferable load is within the acceptable range, indicating that its operation has high flexibility, so the transferable load constraint is expressed as

(18) $$\begin{array}{c} \sum_{t=t_{i,k}^{S_{\text{trans}}}}^{t_{i,k}^{E_{\text{trans}}}}u_{i,k,t}=D_{i,k}^{\text{trans}}, \end{array}$$

where *t*_*i*,*k*_^*S*_trans_^ and *t*_*i*,*k*_^*E*_trans_^, respectively, are the beginning and end dispatch periods of the *k*-th transferable load of MGi.

According to different degrees of importance, the maximum interruptible duration constraint of the interruptible load is implemented. In addition, the maximum and minimum duration constraints are also included in the interruptible load [23], and the constraints are expressed as

(19) $$\begin{array}{c} \sum_{t=t_{i,k}^{S_{\text{inter}}}}^{t_{i,k}^{E_{\text{inter}}}}\left(1-f_{i,k,t}\right)\leq D_{i,k}^{\text{inter}}, \end{array}$$

where *t*_*i*,*k*_^*S*_inter_^ represents the initial operating period of the *k*-th interruptible load in MGi, *t*_*i*,*k*_^*E*_inter_^ represents the end operating period of the *k*-th interruptible load in MGi, and *D*_*i*,*k*_^inter^ represents the *k*-th maximum interruptible duration in MGi in a day.

## 4. Improved Sparrow Algorithm

### 4.1. Basic Sparrow Search Algorithm

The sparrow search algorithm is a swarm intelligence optimization algorithm that simulates the foraging behavior of sparrows. It contains three types of individuals, discoverer, follower, and alerter, and updates their locations according to their own rules.

The location of the discoverer is updated as follows:

(20) $$\begin{array}{c} X_{i,j}^{t+1}=\left\{\begin{array}{c} X_{i,j}^{t}\cdot \;\exp\left(\frac{-i}{\xi \cdot \text{MaxIter}}\right),\;R_{2}<ST,\\ X_{i,j}^{t}+Q\cdot L,\;R_{2}\geq ST, \end{array}\right) \end{array}$$

where *t* represents the current iteration number, *j*=(1,2,…, *d*), and *X*_*i*,*j*_^*t*+1^ represents the position of the *i*-th sparrow of the *t* + 1 generation in the *j*-th dimension, MaxIter represents the maximum number of iterations, *ξ* is a random number in the range of (0,1), *R*_2_ ∈ [0,1] represents the warning value, *ST* ∈ [0.5, 1] represents the safety value, *Q* is a random number and obeys [0,1] normal distribution, and *L* is a row of multidimensional matrix where all elements are 1. If *R*_2_ < *ST*, it means that there are no natural enemies nearby, the search environment is safe, and the discoverer implements an extensive search mode; if *R*_2_ ≥ *ST*, it means that sparrows detect natural enemies, and the entire population adjusts its search strategy and quickly moves to a safe area.

The follower's location update formula is as follows:

(21) $$\begin{array}{c} X_{i,j}^{t+1}=\left\{\begin{array}{c} Q\cdot \;\exp\left(\frac{X_{w,j}^{t}-X_{i,j}^{t}}{i^{2}}\right),\;i>\frac{N}{2},\\ X_{b,j}^{t+1}+\left|X_{i,j}^{t}-X_{b,j}^{t+1}\right|\cdot A^{+}\cdot L,\;i\leq \frac{N}{2}, \end{array}\right) \end{array}$$

where *X*_*w*,*j*_^*t*^ represents the worst position of the sparrow in the *j*-th dimension at the *t*-th iteration, *X*_*b*,*j*_^*t*+1^ represents the best position of the sparrow in the *j*-th dimension at the *t* + 1 iteration, and A is a 1 × *d* matrix with randomly assigned values of 1 or −1 for each element. If *i* > *N*/2, it means that the *i*-th follower did not get food and has low adaptability and needs to fly to other areas to find food to obtain energy. If *i* ≤ *N*/2, it means that the *i*-th follower will randomly select a location nearby *X*_*b*,*j*_^*t*+1^ for foraging.

The position update formula of the alerter is as follows:

(22) $$\begin{array}{c} X_{i,j}^{t+1}=\left\{\begin{array}{c} X_{b,j}^{t}+\beta \cdot \left|X_{i,j}^{t}-X_{b.j}^{t}\right|,\;f_{i}>f_{g},\\ X_{i,j}^{t}+k\cdot \left(\frac{\left|X_{i,j}^{t}-X_{w,j}^{t}\right|}{\left(f_{i}-f_{w}\right)+\varepsilon }\right),\;f_{i}=f_{g}, \end{array}\right) \end{array}$$

where *X*_*b*,*j*_^*t*^ represents the optimal position of the sparrow in the *j*-th dimension at the *t*-th iteration, *β* is the step size control parameter, *k* is a random number within [−1,1], *f*_*i*_ is the fitness value of the current sparrow, *f*_*g*_ represents the current global optimal fitness value, *f*_*w*_ represents the current global worst fitness value, and *ε* is a very small constant to avoid denominator becoming 0. If *f*_*i*_ > *f*_*g*_, it means that the sparrow is on the edge of the population and is easily attacked by natural enemies; if *f*_*i*_=*f*_*g*_, it means that the sparrow is in the center of the population and, due to being aware of the threat of being attacked by natural enemies, approaches other sparrows in time to avoid danger.

### 4.2. Improved Sparrow Algorithm

#### 4.2.1. Initial Population of the Bernoulli Chaotic Map

Chaotic variables are ergodic, which can effectively improve the algorithm's global optimization capability. The chaotic map used in this paper is the Bernoulli equation [24].

(23) $$\begin{array}{c} X_{n+1}=\left\{\begin{array}{c} \frac{X_{n}}{\left(1-\lambda \right)},\;X_{n}\in \left(0,1-\lambda \right],\\ \frac{\left(X_{n}-1+\lambda \right)}{\lambda },\;X_{n}\in \left(1-\lambda ,1\right), \end{array}\right) \end{array}$$

where *λ*=0.3.

#### 4.2.2. Improved Finder Update Formula

Introducing the global optimal solution of the previous generation and the dynamic weight factor *ω* into the discoverer update formula can avoid the algorithm falling into local optimal and improve the convergence speed [25]. The improved formula is as follows:

(24) $$\begin{array}{c} \omega =\frac{\left(\omega_{\min}+\omega_{\max}\right)}{2}+\left(\omega_{\max}-\omega_{\min}\right)\cos\left(\frac{t\pi }{\text{MaxIter}}\right), \end{array}$$

(25) $$\begin{array}{c} X_{i,j}^{t+1}=\left\{\begin{array}{c} X_{i,j}^{t}+\omega \left(f_{j,g}^{t}-X_{i,j}^{t}\right)\cdot \text{rand},\;R_{2}<ST,\\ X_{i,j}^{t}+Q,\;R_{2}\geq ST. \end{array}\right) \end{array}$$

In formula (24), *ω*_max_ and *ω*_min_ are the inertia weights at the beginning and end of the iteration, respectively, *t* is the current iteration number, MaxIter is the maximum number of iterations, and when *ω*_max_=0.95 and *ω*_min_=0.4, the algorithm optimization performance is the best. As the number of iterations increases, the inertia weight will gradually decrease nonlinearly because at the beginning of the iteration, using a larger inertia weight can improve the search ability of the algorithm and at the end of the iteration, using a smaller inertia weight can enhance the development ability of the algorithm.

In formula (25), *f*_*j*,*g*_^*t*^ is the optimal solution of the sparrow in the *j*-th dimension at the *t*-th iteration.

#### 4.2.3. Improved Alerter Update Formula

The improved alerter update formula is shown in equation (26), which means that if the sparrow is in the optimal position, it will randomly fly to any position between the optimal position and the worst position. If the sparrow is not in the optimal position, then it will randomly fly to any position between the current position and the optimal position.

(26) $$\begin{array}{c} X_{i,j}^{t+1}=\left\{\begin{array}{c} X_{b,j}^{t}+\beta \left(X_{i,j}^{t}-X_{b,j}^{t}\right),\;f_{i}\neq f_{g},\\ X_{b,j}^{t}+\beta \left(X_{w,j}^{t}-X_{b,j}^{t}\right),\;f_{i}=f_{g}. \end{array}\right) \end{array}$$

#### 4.2.4. Combining the Cauchy Mutation and Reverse Learning Strategy

Gaussian mutation has a weak ability to guide individuals out of better local solutions, which is not conducive to global convergence; therefore, this paper uses the Cauchy mutation [26–28]. The Cauchy mutation comes from the Cauchy distribution, and the standard Cauchy distribution function formula is as follows:

(27) $$\begin{array}{c} f\left(x\right)=\frac{1}{\pi }\left(\frac{1}{x^{2}+1}\right). \end{array}$$

Introducing the Cauchy mutation into the target position update method and exerting the perturbation ability of the Cauchy operator can improve the global optimization ability of the algorithm.

(28) $$\begin{array}{c} X_{i,j}^{t+1}=X_{b,j}\left(t\right)+\text{cauchy}\left(0,1\right)⊕X_{b,j}\left(t\right), \end{array}$$

where cauchy(0,1) is the standard Cauchy distribution. The Cauchy distribution random variable generating function is *η*=tan[(*ξ* − 0.5)*π*].

Reverse learning can effectively improve the efficiency of the algorithm in solving the global optimum. The solution idea is: in the search process, based on the current solution, the reverse learning strategy is used to calculate the reverse solution relative to the center, and then, a better solution is selected after the corresponding comparative evaluation, thereby improving the global optimization capability of the algorithm, and the calculation formula is as follows:

(29) $$\begin{array}{c} X_{b,j}^{\prime }\left(t\right)=ub+r⊕\left(lb-X_{b,j}\left(t\right)\right), \end{array}$$

(30) $$\begin{array}{c} X_{i,j}^{t+1}=X_{b,j}^{\prime }\left(t\right)+k_{1}⊕\left(X_{b,j}\left(t\right)-X_{b,j}^{\prime }\left(t\right)\right), \end{array}$$

where *X*_*b*,*j*_′(*t*) is the reverse solution of the optimal solution of the *t*-th generation, *ub*, *lb* are the upper and lower bounds, *r* is a 1^*∗*^*d* random number matrix, which obeys the (0, 1) standard uniform distribution, and *k*_1_ represents the information exchange control parameters. The formula is as follows:

(31) $$\begin{array}{c} k_{1}={\left(\text{MaxIter}-\frac{t}{\text{MaxIter}}\right)}^{t}. \end{array}$$

The reverse learning strategy can expand the global optimization ability of the algorithm, and the Cauchy mutation strategy can improve the algorithm to avoid falling into the local optimal solution. Therefore, in order to further improve the algorithm optimization performance, the Cauchy mutation strategy and the reverse learning strategy are exchanged under the condition of selection probability *P*_*s*_, and the target position is dynamically updated. The calculation formula is as follows:

(32) $$\begin{array}{c} P_{s}=-\exp\;{\left(1-\frac{t}{\text{MaxIter}}\right)}^{20}+\theta , \end{array}$$

where *θ* is the adjustment parameter, and its value can be 0.05.

The process of selecting strategy 1 is as follows:

1. If rand < *P*_*s*_
2. Select the reverse learning strategy of formulas (29)–(31) to update the position
3. If rand ≥ *P*_*s*_
4. Select the Cauchy mutation strategy of formula (28) to update the target position

The Cauchy perturbation strategy and reverse learning strategy can improve the ability of the algorithm to jump out of local space; however, it is impossible to compare whether the fitness value of the new position obtained after these two disturbance strategies is better than the fitness value of the previous position. Therefore, the greedy rule is added to compare the fitness value of the new position after the disturbance mutation with the fitness value of the previous position. If the fitness value of the new location is better than the fitness value of the old location, the location is updated; otherwise, it is not updated.

The process of selecting strategy 2 is as follows:

1. If *f*(*X*_*i*,*j*_^*t*+1^) < *f*(*X*_*b*,*j*_)
2. *X*_*b*,*j*_=*X*_*i*,*j*_^*t*+1^
3. If *f*(*X*_*i*,*j*_^*t*+1^) ≥ *f*(*X*_*b*,*j*_)
4. *X*_*b*,*j*_=*X*_*b*,*j*_

#### 4.2.5. The Steps of the Algorithm

The steps of the chaos sparrow algorithm are as follows:

1. Initialize the parameters, and use the Bernoulli chaotic map of equation (23) to initialize the sparrow population
2. Calculate the fitness value of each sparrow and sort it to find the current optimal and worst fitness value individual
3. According to equations (25), (21), and (26), the positions of the finder, follower, and alerter are updated, respectively
4. According to selection strategy 1, the current optimal solution is disturbed and a new solution is generated
5. According to strategy 2, compare the pros and cons of the fitness values before and after the disturbance to determine whether the location is updated
6. Iteration termination judgment: if it meets the iteration termination condition, then jump out of the loop and output the optimal result; if not, then jump to step (2) and continue the iteration until it meets the iteration termination condition and output the optimal result.

## 5. Function Test

### 5.1. Parameter Setting

Based on 12 benchmark test functions, we compare the performance of the improved sparrow algorithm, sparrow algorithm, particle swarm algorithm, chaotic particle swarm algorithm, and genetic algorithm. The test function is shown in Table 1, and the parameters of each algorithm are shown in Table 2. The parameter selection was based on the parameters used by the original author in the article or the parameters widely used by various researchers. The simulation is written and completed by MATLAB2018a. In the test, the population size of each algorithm is set to 50, the number of iterations is set to 400, and each algorithm runs independently 50 times.

### 5.2. Comparison and Analysis of Algorithm Performance Results

The average values and standard deviations obtained by optimizing 12 benchmark test functions by five algorithms are shown in Table 3. It can be seen that, for the unimodal function F1–F7, the ISSA algorithm has the best optimization effect, and the average value and standard deviation reach the global optimal solution. For the multimodal function F8–F12, ISSA algorithm and SSA algorithm have the same optimization effect and are better than PSO, CPSO, and GA algorithm. When solving F8 and F10, ISSA and SSA can reach the theoretical optimal solution stably. When solving F9, F11, and F12, although ISSA and SSA cannot converge to the theoretical optimal solution, their convergence effect and convergence accuracy are better than those of PSO, CPSO, and GA.

### 5.3. Comparison and Analysis of Algorithm Convergence Curves

**Figure 2:**
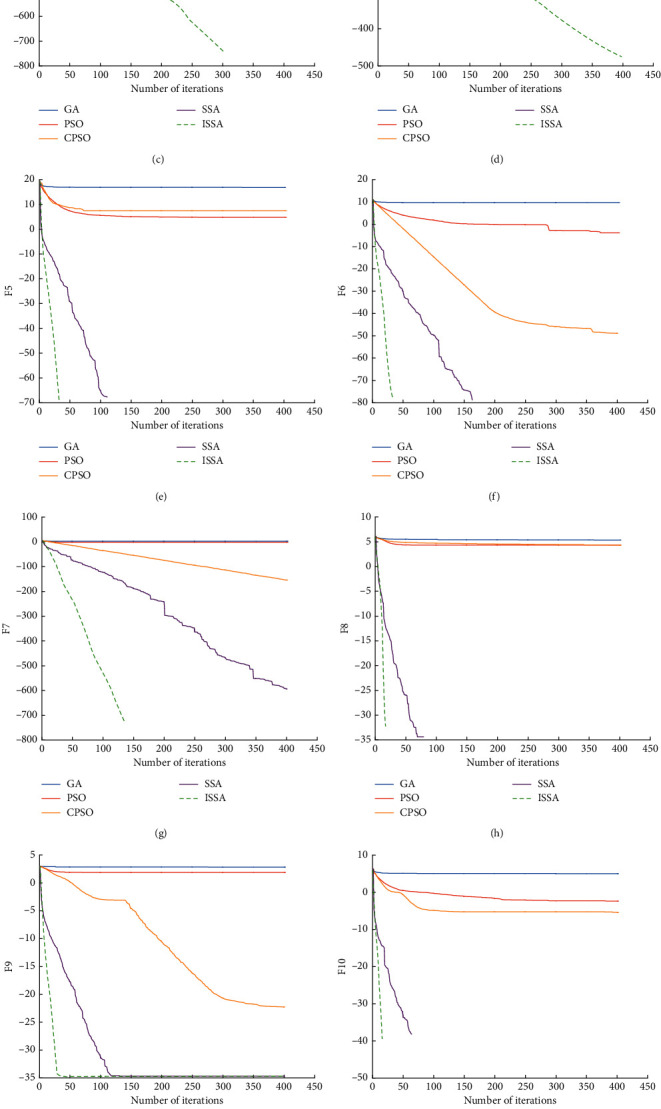
Convergence curve of algorithm. (a) F1. (b) F2. (c) F3. (d) F4. (e) F5. (f) F6. (g) F7. (h) F8. (i) F9. (j) F10. (k) F11. (m) F12.

The convergence curve obtained by optimizing 12 benchmark test functions by five algorithms is shown in Figure 2. It can be seen that when solving different test functions, the convergence speed of ISSA is better than SSA, PSO, CPSO, and GA algorithms, and it has better convergence speed and convergence accuracy. The horizontal axis represents update algebra, and the vertical axis represents the logarithm of fitness value log.

## 6. Case Analysis

### 6.1. Basic Data

**Figure 3:**
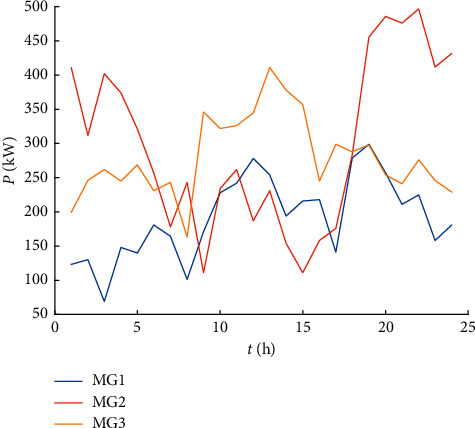
Renewable energy output.

**Figure 4:**
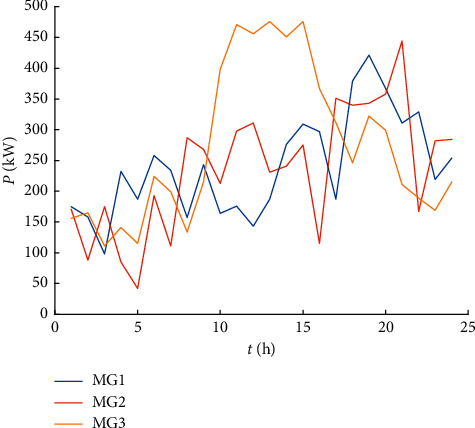
The original load of each microgrid.

This article takes the microgrid cluster system constructed by the three microgrids shown in Figure 1 as an example. The renewable energy output of each microgrid is shown in Figure 3, and the load curve is shown in Figure 4. The power of the controllable loads is 25 kW, and the dispatch compensation prices for the shiftable load, the transferable load, and the interruptible load, respectively, are 0.05 Yuan/kWh, 0.08 Yuan/kWh, and 0.3 Yuan/kWh. The initial operating period and allowable scheduling period of the controllable loads are shown in Table 4. The rated capacity of the microgrid energy storage system is 300 kWh, the maximum allowable charge and discharge power is 100 kW, and the maximum and small states of charge are divided into 0.9 and 0.2. The purchase and sale price of the power distribution network is shown in Table 5. The scheduling period of the calculation example is 24 hours a day, divided into 24 time periods, and the scheduling time is selected as 00 : 00–24 : 00.

### 6.2. Analysis of the Results of Electricity Trading between Microgrids

The electric energy exchange between microgrids is shown in Table 6. It can be seen that when the market is in equilibrium, electricity can be purchased and sold in various periods of time through the trade of electricity between microgrids.

It can be seen from Table 6 that when controllable loads participate in dispatching, at 14 : 00, 15 : 00, and 17 : 00, since the power generation of the microgrid cluster cannot meet the load demand, the power interaction between the microgrids in the cluster cannot be carried out, and electricity needs to be purchased from the power distribution network. From 10 : 00 to 13 : 00, MG1 sells electricity to MG2 that has insufficient power at the target time due to its own renewable energy output to meet its own load demand while still having electricity surplus. During the time periods of 08 : 00–09 : 00, 11 : 00–12 : 00, and 18 : 00, MG2 cannot meet its own load demand due to its insufficient output. Therefore, MG2 needs to purchase electricity from MG1 or MG3 that has too much power at the target time. During the time period from 19 : 00 to 24 : 00, MG3 sells electricity to the MG1 whose power is insufficient at the target time due to its own renewable energy output to meet its own load demand while still having electricity surplus.

### 6.3. Analysis of the Results of Electric Energy Trading between the Microgrid and Power Distribution Network

The electric energy exchange between the microgrid and the power distribution network is shown in Table 7. It can be seen that when MG1, MG2, and MG3 exchange electric energy, the interactive electric energy between each microgrid and the power distribution network can be reduced.

It can be seen from Table 7 that when controllable loads participate in the dispatch, during the time period of 14 : 00–18 : 00, MG1 cannot meet its own needs due to its insufficient output; therefore, it needs to purchase electricity from the power distribution network. During the time period of 01 : 00–05 : 00 and 19 : 00–24 : 00, MG2 has been selling electric energy to the power distribution network due to its overcapacity. During the time period of 10 : 00–17 : 00, MG3 cannot meet its own needs due to its insufficient output, and needs to purchase electricity from the power distribution network to meet its own needs. It can be seen from the abovementioned phenomenon that electricity trading between microgrids can completely eliminate excess renewable energy and balance the supply and demand of the system.

### 6.4. Analysis of the Results before and after Controllable Loads

**Figure 5:**
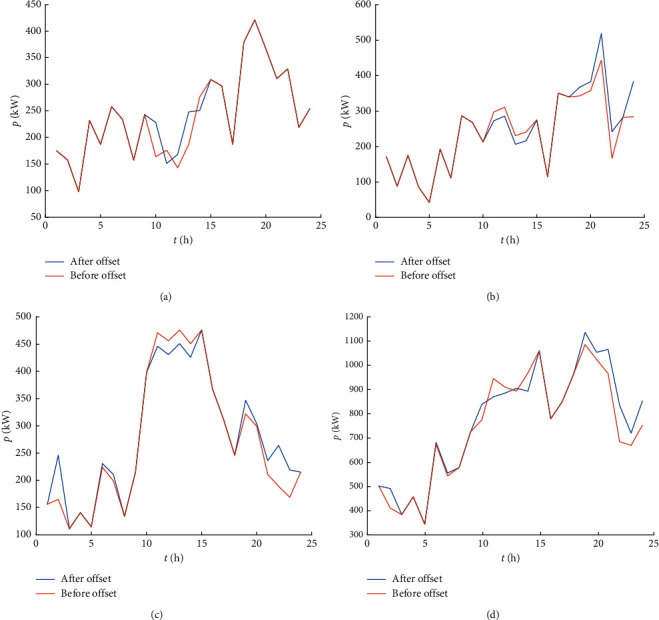
Before and after load shift. (a) MG1; (b) MG2; (c) MG3; and (d) microgrid cluster.

The controllable load scheduling results are shown in Figure 5. It can be seen from Figure (a–c) that the overall loads of MG1 increase from 09 : 00 to 15 : 00, the loads of MG2 decrease from 10 : 00 to 15 : 00, and the loads increase from 18 : 00 to 24 : 00, the loads of MG3 increase during the time period of 00 : 00–08 : 00 and 18 : 00–24 : 00, and the loads decrease from 09 : 00 to 15 : 00. The total loads of the microgrid cluster can be seen from Figure (d), after adding the demand-side controllable loads schedule; the loads in the low period of 23 : 00–07 : 00 increase, and the loads in the peak period of 11 : 00–15 : 00 decreases. The peak-valley difference of the loads is reduced.

### 6.5. Economic Analysis of the Microgrid Cluster

The costs of the microgrid cluster in the two cases where the microgrid cluster has demand-side controllable loads participating in the dispatch and the uncontrollable loads participating in the dispatch are compared, as shown in Table 8.

It can be seen from Table 8 that, in the two cases where there are controllable loads participating in the dispatch and the uncontrollable load participating in the dispatch, the cost change of energy storage system is relatively small. When controllable loads are involved in dispatching, the total cost of MG1 is 1067.4864 Yuan, the total revenue of MG2 is 520.6545 Yuan, and the total cost of MG3 is 107.4852 Yuan. Compared with when there is no controllable load involved in dispatching, the economic cost of MG1 is reduced by 87.0533 Yuan, the revenue of MG2 is reduced by 33.1091 Yuan, and the revenue of MG3 is increased by 109.9848 Yuan. Therefore, the economic cost of the entire microgrid cluster is reduced by 163.9284 Yuan. It can be seen that when the controllable loads participate in dispatching, the microgrid cluster can effectively reduce the operating cost of the system.

### 6.6. Analysis of Optimization Algorithm Results

This paper proposes a chaotic sparrow search algorithm. This algorithm is used to optimize the operating cost of the microgrid cluster system, and it compares with sparrow algorithm, particle swarm algorithm, chaotic particle swarm algorithm, and genetic algorithm. The basic parameters of the algorithm are shown in Table 2.

**Figure 6:**
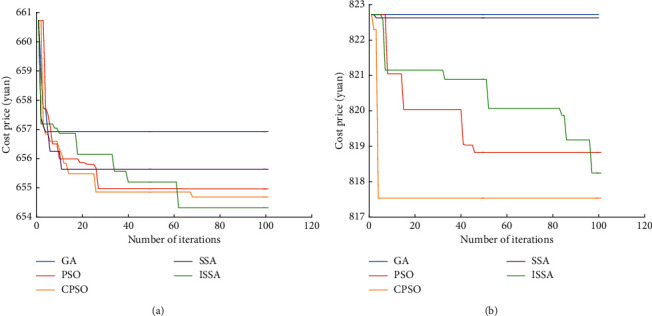
Algorithm comparison. (a) Controllable loads; (b) no controllable loads.

It can be seen from Figure 6 that the objective function values of the five algorithms of GA, PSO, CPSO, SSA, and ISSA decrease continuously and become stable as the number of iterations increases, indicating that these five algorithms are all moving towards the optimal cost. It can be found by comparing GA, PSO, CPSO, SSA, and ISSA that when the demand-side controllable loads participate in dispatching, the number of iterations for ISSA algorithm to converge to the optimal solution is similar to CPSO algorithm, and its optimal solution is better than the convergence solutions of GA, PSO, CPSO, and SSA algorithms. When there is no demand-side controllable load participating in dispatching, the number of iterations for ISSA algorithm to converge to the optimal solution is higher than GA, PSO, CPSO, and SSA, and its optimal solution is better than the convergence solution of GA, PSO, and SSA algorithm.

## 7. Conclusions

This paper sets the optimization goal as the operating cost of the microgrid cluster system, constructs the microgrid cluster operation optimization model considering demand-side response, and discusses the costs of the microgrid cluster with and without controllable loads dispatch. Then a chaos sparrow search algorithm based on Bernoulli chaotic mapping, dynamic adaptive weighting, Cauchy mutation, and reverse learning is constructed, which is used to optimize the operating cost of the microgrid cluster system and compare with the optimized results of sparrow algorithm, particle swarm algorithm, chaotic particle swarm algorithm, and genetic algorithm.

1. Through the proposed strategy, electric energy transactions between microgrids and between microgrids and power distribution networks are realized. On the one hand, the impact caused by the connection between the microgrid cluster and power distribution networks is minimized, and on the other hand, it ensures the safety and stability of system operation.
2. When there is demand-side controllable loads participating in dispatching, on the one hand, the level of energy mutual benefit between microgrids is improved, and the excess renewable energy can be completely absorbed; on the other hand, due to the controllable loads participating in dispatching, the operating cost of the microgrid cluster dropped from 818.2455 Yuan to 654.3171 Yuan, the cost was reduced by 163.9284 Yuan, and the overall benefit has increased by nearly 20%, which effectively improved the economic benefits of the microgrid cluster.
3. This paper proposes a chaos sparrow search algorithm based on Bernoulli chaotic mapping, dynamic adaptive weighting, Cauchy mutation, and reverse learning. Based on 12 benchmark test functions, it shows that ISSA algorithm is superior to SSA, PSO, CPSO, and GA algorithms in terms of solution quality, which proves the effectiveness of the algorithm improvement. At the same time, ISSA algorithm has better performance when solving microgrid cluster optimization problems. The global optimization ability is stronger than SSA, PSO, CPSO, and GA algorithms, and the optimization cost is lower than SSA, PSO, CPSO, and GA algorithms. In future works, the ISSA algorithm will be applied to other fields, such as energy storage capacity configuration optimization, and face recognition. It can also be combined with other algorithms to form a hybrid algorithm to improve algorithm performance.

## Figures and Tables

**Table 1:** Test function.

Function expression	Dimension	Range	Optimal value
*F*_1_(*x*)=∑_*i*=1_^*n*^*x*_*i*_^2^	30	[−100,100]	0
*F*_2_(*x*)=∑_*i*=1_^*n*^|*x*_*i*_|+∏_*i*=1_^*n*^|*x*_*i*_|	30	[−10,10]	0
*F*_3_(*x*)=∑_*i*=1_^*n*^(∑_*j*−1_^*i*^*x*_*j*_)^2^	30	[−100,100]	0
*F*_4_(*x*)=max_*i*_{|*x*_*i*_|, 1 ≤ *i* ≤ *n*}	30	[−100,100]	0
*F*_5_(*x*)=∑_*i*=1_^*n*−1^[100(*x*_*i*+1_ − *x*_*i*_^2^)^2^+(*x*_*i*_ − 1)^2^]	30	[−30,30]	0
*F*_6_(*x*)=∑_*i*=1_^*n*^([*x*_*i*_+0.5])^2^	30	[−100,100]	0
*F*_7_(*x*)=∑_*i*=1_^*n*^*ix*_*i*_^4^+random[0,1)	30	[−1.28, 1.28]	0
*F*_8_(*x*)=∑_*i*=1_^*n*^[*x*_*i*_^2^ − 10 cos(2*πx*_*i*_)+10]	30	[−5.12, 5.12]	0
$F_{9}\left(x\right)=-20\;\exp\left(-0.2\sqrt{\left(1/n\right)\sum_{i=1}^{n}x_{i}^{2}}\right)-\exp\left(\left(1/n\right)\sum_{i=1}^{n}\cos\left(2\pi x_{i}\right)\right)+20+e$	30	[−32,32]	0
$F_{10}\left(x\right)=\left(1/4000\right)\sum_{i=1}^{n}x_{i}^{2}-\prod_{i=1}^{n}\cos\left(x_{i}/\sqrt{i}\right)+1$	30	[−600,600]	0
$\begin{array}{c} F_{11}\left(x\right)=\left(\pi /n\right)\left\{10\;\sin\left(\pi y_{1}\right)+\sum_{i=1}^{n-1}{\left(y_{i}-1\right)}^{2}\left[1+\;\sin^{2}\left(\pi y_{i+1}\right)\right]+{\left(y_{n}-1\right)}^{2}\right\}+\sum_{i=1}^{n}u\left(x_{i},10,100,4\right)\\ y_{i}=1+\left(x_{i}+1/4\right)u\left(x_{i},a,k,m\right)=\left\{\begin{array}{c} k{\left(x_{i}-a\right)}^{m},x_{i}>a\\ 0,-a<x_{i}<a\\ k{\left(-x_{i}-a\right)}^{m},x_{i}<-a \end{array}\right) \end{array}$	30	[−50,50]	0
*F*_12_(*x*)=0.1{sin^2^(3*πx*_1_)+∑_*i*=1_^*n*^(*x*_*i*_ − 1)^2^[1+ sin^2^(3*πx*_*i*_+1)]+(*x*_*n*_ − 1)^2^[1+ sin^2^(2*πx*_*n*_)]}+∑_*i*=1_^*n*^*u*(*x*_*i*_, 5,100,4)	30	[−50,50]	0

**Table 2:** Parameter settings of the algorithm.

Algorithm	Parameter settings
GA	PX=0.65, PM=0.05
PSO	*c*1=*c*2=1, *w*=0.6
CPSO	*c*1=*c*2=1, *w*=0.6
SSA	PD=20%, *R*_2_=0.8, SD=10%
ISSA	PD=20%, *R*_2_=0.8, SD=10%, *θ*=0.05

**Table 3:** Comparison results on test functions.

F	Statistics	GA	PSO	CPSO	SSA	ISSA
F1	Ave	1.5885*e* + 04	0.0557	6.3925*e* − 40	6.9803*e* − 134	0
	Std	3.5365*e* + 03	0.2323	1.0020*e* − 39	4.9358*e* − 133	0
F2	Ave	50.2661	7.2639	2.3988*e* − 20	3.5405*e* − 71	1.0603*e* − 219
	Std	8.8456	5.1873	1.5270*e* − 20	2.5035*e* − 70	0
F3	Ave	3.1834*e* + 04	1.8163*e* + 03	22.9941	4.3294*e* − 107	0
	Std	9.0644*e* + 03	1.4864*e* + 03	81.9827	2.3121*e* − 106	0
F4	Ave	53.4554	11.8072	11.4627	4.0192*e* − 76	6.4391*e* − 208
	Std	5.7831	3.7901	3.3974	2.8420*e* − 75	0
F5	Ave	2.0631*e* + 07	114.0382	1.7179*e* + 03	0	0
	Std	8.4778*e* + 06	202.1385	1.1891	0	0
F6	Ave	1.6195*e* + 04	0.0209	5.7585*e* − 22	0	0
	Std	4.4542*e* + 03	0.1128	1.6550*e* − 21	0	0
F7	Ave	8.7427	0.1611	9.4820*e* − 68	1.4052*e* − 258	0
	Std	4.5183	0.6440	5.2353*e* − 67	0	0
F8	Ave	210.7998	76.9175	77.3060	0	0
	Std	28.4726	23.9501	24.3339	0	0
F9	Ave	17.0921	6.5281	2.1541*e* − 10	8.8818*e* − 16	8.8818*e* − 16
	Std	0.7411	1.3035	9.4122*e* − 20	0	0
F10	Ave	151.2605	0.0943	0.0043	0	0
	Std	33.9782	0.0976	0.0069	0	0
F11	Ave	1.4287*e* + 07	5.5806	1.4205*e* − 31	1.5705*e* − 32	1.5705*e* − 32
	Std	1.0079*e* + 07	3.6888	1.2059*e* − 31	5.5294*e* − 48	5.5294*e* − 48
F12	Ave	5.6904*e* + 07	25.0058	3.9269*e* − 31	1.3498*e* − 32	1.3498*e* − 32
	Std	3.0359*e* + 07	10.7048	5.9462*e* − 31	1.1059*e* − 47	1.1059*e* − 47

**Table 4:** Controllable loads parameters.

Time period	Shiftable load	Transferable load	Interruptible load
Initial operating period	11 : 00–15 : 00	19 : 00–22 : 00	10 : 00–21 : 00
Scheduleable period	11 : 00–22 : 00	00 : 00–24 : 00	10 : 00–21 : 00

**Table 5:** Time-of-use price.

Transaction form	Timetable		Electricity price (Yuan/(kwh))
Sell electricity	Peak time	10 : 00∼15 : 00 18 : 00∼21 : 00	1.2
	Normal time	07 : 00∼10 : 00 15 : 00∼18 : 00 21 : 00∼23 : 00	0.8
	Valley time	23 : 00∼07 : 00	0.4
Purchase electricity	0 : 00∼24 : 00		0.39

**Table 6:** Transaction electricity between microgrids.

Time	Transaction power (kwh) (controllable load)			Transaction power (kwh) (no controllable load)		
	MG1	MG2	MG3	MG1	MG2	MG3
1	57.0021	−49.0573	−7.9448	56.9146	−48.9979	−7.9167
2	30.5244	−33.5049	2.9805	30.4912	−22.5127	−7.9785
3	30.2046	−18.1475	−12.0571	30.1978	−18.1446	−12.0472
4	84.6933	−62.3520	−22.3413	84.3783	−62.1016	−22.2767
5	47.1775	−30.4015	−16.7760	47.8766	−30.8375	−17.0391
6	63.5587	−63.7779	0.2192	69.7560	−63.0511	−6.7049
7	70.0852	−47.4771	−22.6081	70.3143	−42.3144	−27.9999
8	16.1676	12.6786	−28.8465	15.9683	12.7390	−28.7072
9	41.2814	89.3333	−130.6147	41.0727	89.6763	−130.7490
10	−0.0712	−21.2586	21.3298	−57.9441	−18.2874	76.2315
11	−91.1144	7.7568	81.3576	−65.6829	13.3159	52.3669
12	−110.2000	59.1433	51.0568	−134.7312	71.2757	63.4555
13	−6.6502	−25.2724	31.9226	−66.1035	0.9295	65.1741
14	0	0	0	0	0	0
15	0	0	0	0	0	0
16	16.9902	−43.1368	26.1467	17.3261	−43.7299	26.4038
17	0	0	0	0	0	0
18	26.8221	14.9465	−41.7686	28.1474	15.1149	−43.2623
19	71.8018	−71.1782	−0.6236	97.1128	−116.1794	19.0666
20	60.0222	−60.0180	−0.0042	93.7188	−131.3682	37.6494
21	49.2324	−6.0715	−43.1609	64.2092	−33.1416	−31.1416
22	52.5377	−43.5587	−8.9790	104.5194	−82.3977	−22.1217
23	58.5305	−47.8465	−10.6840	58.7109	−37.1341	−21.5768
24	68.5343	−49.9633	−18.5710	69.4401	−62.4023	−7.0378

**Table 7:** Transaction electricity between the microgrid and power distribution network.

Time	Transaction power (kwh) (controllable load)			Transaction power (kwh) (no controllable load)		
	MG1	MG2	MG3	MG1	MG2	MG3
1	0	185.9288	30.1110	−7.1054*e* − 15	185.8260	30.0244
2	0	188.2493	0	0	198.6898	70.4152
3	0	207.5529	137.8976	0	206.9558	137.4090
4	0	226.2737	81.0761	0	225.9525	81.0524
5	0	247.9922	136.8463	0	247.2298	136.6060
6	−13.6186	0	−0.0470	−7.6627	−7.1054*e* − 15	0
7	0	19.1792	9.1329	0	23.7368	15.7069
8	−39.8650	−31.2620	0	−40.3733	−32.2086	0
9	−31.2095	−67.5377	0	−31.2378	−68.2033	0
10	0	0	−54.9251	5.7801	1.8242	0
11	1.4211*e* − 14	−3.3936	−36.4689	0	−23.6277	−92.9195
12	0	−39.3185	−33.9426	0	−53.7049	−47.8125
13	0	0	−7.0099	0.6195	0	0
14	−56.9322	−61.8237	−47.6179	−82.3522	−87.8662	−73.1322
15	−93.0122	−164.1142	−118.7230	−93.4173	−164.3346	−118.9401
16	−62.0762	0	−95.5308	−62.2867	0	−94.9206
17	−45.9460	−174.8343	−12.8887	−46.5703	−173.2011	−11.2288
18	−73.2103	−40.7362	0	−71.2530	−38.2621	0
19	0	67.3300	0.5899	−22.2530	0	−4.3334
20	0	93.5763	0.0065	−13.1720	0	−5.2915
21	0	1.5837	11.2581	−33.3161	0	0
22	0	263.2646	54.2685	0	248.6835	66.7652
23	0	84.8327	18.9430	0	95.0723	55.2418
24	0	0.1539	0.0572	0	89.9630	10.1462

**Table 8:** Operating costs of the microgrid cluster.

Types	Controllable load			No controllable load		
	MG1	MG2	MG3	MG1	MG2	MG3
ESS	2.6302	2.5927	3.0154	3.7120	5.1049	3.1601
Load_shift_	2.5000	2.5000	2.5000	0	0	0
Load_trans_	8	8	8	0	0	0
Load_inter_	30	0	6	0	0	0
MG and MG	637.1300	−489.1636	−147.9664	655.6866	−609.4750	−46.2116
MG and DN	387.2262	−44.5836	235.9362	495.1411	50.6059	260.5215
Total cost	1067.4864	−520.6545	107.4852	1154.5397	−553.7642	217.4700
		654.3171			818.2455	

## Data Availability

The data used to support the findings of this study are available from the corresponding author upon request.

## Abbreviation

BD: Battery degradation
CL: Controllable load
MN: Microgrid network
DN: Distribution network
ch: Charge
dis: Discharge
ESS: Energy storage battery
IL: Important load
RE: Renewable energy
EB: Electricity buy
ES: Electricity sell
Min: minimum
max: Maximum.
